# Experimental Study on Reducing the Degradation of Mechanical Properties in Huntite-Hydromagnesite-Filled Flame-Retardant Polymer Composites Containing Calcite and Clay for Engineering Applications

**DOI:** 10.3390/ma19214469

**Published:** 2026-10-23

**Authors:** Gül Yılmaz Atay, Jacek Lukasz Wilk-Jakubowski, Valentyna Loboichenko

**Affiliations:** 1 Faculty of Engineering and Architecture, Department of Materials Science and Engineering, Izmir Katip Çelebi Univeristy, 35620 İzmir, Turkey; hgulyilmaz@gmail.com; 2 Department of Information Systems, Kielce University of Technology, 7 Tysiąclecia Państwa Polskiego Ave., 25-314 Kielce, Poland; 3 Departamento de Ingeniería Energética, Escuela Técnica Superior de Ingeniería, Universidad de Sevilla, Camino de los Descubrimientos s/n., 41092 Sevilla, Spain; 4 Department of Civil Security, Lutsk National Technical University, Lvivska St., 75, 43000 Lutsk, Ukraine

**Keywords:** electrical engineering, polymer composite, flame retardant, mechanical properties, huntite hydromagnesite, calcite, clay

## Abstract

In this work, the possibility of ensuring sufficient fire resistance while preserving the mechanical properties of composites based on polypropylene with the use of hybrid systems of mineral fillers, including huntite/hydromagnesite, calcite, and clay, was studied. Composites with different polypropylene/mineral filler ratios were produced using twin-screw extruder and injection molding methods. The tensile properties, Limiting Oxygen Index, UL-94 vertical combustion performance, and fracture surface morphologies of the samples were evaluated. The results showed that the elastic modulus increased with increasing total mineral filler ratio, while the maximum tensile stress and elongation at break decreased. Using huntite/hydromagnesite in a hybrid with calcite and clay at the same total filler ratio resulted in a partial improvement in mechanical properties compared to composites containing only huntite/hydromagnesite. The highest flame-retardancy performance was obtained in composites containing 70% total mineral filler; all samples in this group met the UL-94 V0 class, and the highest Limiting Oxygen Index value of 42.6% was determined in the sample with a polypropylene:huntite/hydromagnesite:clay ratio of 30%:60%:10%. The developed composites exhibit the highest fire-resistance class. The findings reveal that huntite/hydromagnesite/calcite/clay hybrid mineral systems are a promising alternative that can contribute to balancing mechanical performance while providing halogen-free flame retardancy in polypropylene composites.

## 1. Introduction

It is a well-known fact that fires lead to loss of life and injuries due to high heat or suffocating gases, cause extensive damage to structures, and disrupt nature and the ecological balance. Furthermore, fire is no longer just a security issue but has become a direct issue of economic survival. Because the data proves that fire safety is a vital existential issue for the operational continuity of companies and the national economy [1,2,3].

For a material to be considered fire-resistant, it must be subjected to a certain period of time and standard heat intensity conditions, in which the material does not structurally collapse, does not allow heat transfer, and does not reach a temperature higher than a certain temperature on the side far from the fire [4,5,6]. Fire-resistant materials that meet these conditions are generally grouped into two classes: halogenated and halogen-free. Halogenated flame retardants achieve their effect by interrupting the combustion process, which leads to an increase in the amount of smoke produced. Brominated and chlorinated flame-retardant systems stop the combustion reaction while releasing hydrobromic or hydrochloric acid as byproducts into the atmosphere and emitting toxic gases (especially CO) into the environment. Carbon monoxide is a colorless and odorless gas that is not detected in the initial stages. It rapidly combines with hemoglobin in the blood to form carboxyhemoglobin [7,8,9]. When inhaled, it blocks vital oxygen in the blood, causing sudden fainting. This can lead to human deaths during fire suppression/retardation. In fact, the use of such halogen-containing substances is banned in many European countries [10,11,12].

Halogen-free substances are generally endothermic substances that decompose when a fire starts. During the decomposition reaction, heat is removed from the combustion surface, the burning substrate cools, and consequently, further spread of flames and smoke formation is limited [13,14,15]. If gaseous by-products are released as a result of the decomposition reaction, they lead to a greater spread of heat load in the immediate vicinity of the fire. Gaseous by-products also reduce the oxygen concentration in the combustion area, thus reducing flame spread [16,17]. Among the most commonly used halogen-free substances are inorganic minerals containing hydroxides and bicarbonates, which undergo a compositional change at temperatures of 200–400 °C, releasing water and carbon dioxide. The released water dilutes the combustible gas and prevents the oxygen necessary for combustion. Due to the cooling effect in the environment, the flame-retardant substance coats the combustible surface by forming a ceramic structure on the combustible surface, which prevents the flame and heat from increasing and inhibits the progression of the combustion process [18]. As a result, the flame-retardant substance reduces the heat, fuel, and oxygen required for the fire. At the same time, it slows down the continuation of the fire by preventing these three from coming into contact with each other. It prevents easy ignition, prevents the spread of flame, reduces the rate of heat dissipation, and reduces the amount of smoke emitted [19,20].

In our previous studies, we produced excellent flame-retardant composites by using huntite and hydromagnesite minerals, which operate based on this principle, within polymer composites. However, due to the high loading amount of these minerals in the composite, hardening and brittleness generally occurred in the products. While an increase in elastic modulus was observed, a decrease in tensile strength and especially toughness was observed [19,20]. In one of our studies, when the filler loading amount was increased from 49.75% to 64.06%, the tensile strength decreased from 13.73 MPa to 11.32 MPa, and the elongation at break decreased from 592% to 193% [20].

Studies have also been conducted using various auxiliary minerals to mitigate or completely eliminate the degradation associated with huntite hydromagnesite. These studies revealed that the final properties of the composite are influenced not only by the physical and chemical characteristics of the auxiliary materials but also by properties such as particle size, particle shape, and surface energy. Slight improvements in mechanical properties were observed in these studies [21,22].

This study advances the understanding of polymer-based composites incorporating hybrid mineral systems (huntite/hydromagnesite, calcite, and clay). Specifically, the ability of such polypropylene-based composites to provide satisfactory mechanical properties while maintaining fire resistance has been demonstrated. In particular, the viability of the H/CA/CL system, providing a V-0 flammability class and an oxygen index (LOI) of >40%, has been demonstrated, given certain structural limitations (maximum component content of 70 wt.%), which constitutes the original contribution of this work.

In this study, we aimed to both maintain the high flame-retardant property obtained and repair the deteriorating mechanical properties by using huntite and hydromagnesite along with other additives. It is believed that different mineral types have different effects on the composite. For example, it is possible for different minerals to influence the polymer matrix-filler interaction and improve interfacial adhesion at the boundary between the polymer matrix and the filler. It is thought that the more compatible interfaces obtained in this way have a supportive effect on resistance to crack formation and propagation. The degree of interfacial compatibility depends on factors such as the structure of the filler, surface properties, and reactive regions within the polymer matrix [23,24,25]. In this study, calcite (CA) and clay (CL) were selected as auxiliary additives to accompany the huntite hydromagnesite (H) mineral. Each additive was designed to perform different functions within the composite. The spherical grain structure of calcite will improve the ductility of the composite. Conversely, the platy grain structure of huntite and hydromagnesite is known to increase brittleness, as this structure leads to incompatibility between the matrix and the additive [19]. Clay, on the other hand, is expected to increase the strength of the composite due to its fine-grained structure and very small grain size. In addition, the particle size distribution of clay is very wide. Consequently, a more compatible interface can be obtained because it can more easily fill the voids of varying sizes at the matrix-additive interface. In practice, additive materials used in different ratios were first processed in a polypropylene matrix using a twin-screw extruder at 185 °C and 190 rpm, and samples were produced. The thermal properties of the produced composites were determined by thermogravimetric analysis (TGA). FTIR (Fourier Transform Infrared) spectroscopy was used to examine the bonds and interactions between the components of the composite materials. The structural and morphological properties of the composites were characterized by scanning electron microscopy (SEM) analysis. The flame-retardant performance of the samples was observed according to the Limiting Oxygen Index (LOI) and UL-94 vertical flammability tests. Tensile tests were applied to the samples to determine their mechanical properties.

The developed polymer composites with enhanced fire resistance and good mechanical properties may find applications in the manufacture of electrical equipment housings, wire insulation, or protective enclosures for electronic components operating under demanding thermal conditions. An example of an engineering application in the context of the structural material under consideration could be an acoustic fire extinguisher, particularly the construction of its working components, and specifically its waveguide, which may be exposed to high temperatures and thermal radiation [1,4,5]. In devices of this type, the material must withstand direct thermal radiation during flame suppression while maintaining structural stiffness, stable sound-wave propagation, and dimensional stability, without the emission of toxic gases.

In practice, in the case of hybrid composites, the released water vapor and carbon dioxide locally dilute the flammable gases and cool the structure, while the ceramic layer formed on the surface prevents oxygen from reaching the matrix. The mechanical response of the material also remains important, as the structural material exhibits high stiffness, as confirmed by the high elastic modulus values obtained in tensile and flexural tests. The UL classification and LOI value determined experimentally make these materials a promising candidate for applications involving thermal exposure in the immediate vicinity of a fire.

## 2. Materials and Methods

The carrier polymer matrix was thermoplastic polypropylene (PP) coded Petoplen EH251 with a density of 0.905 g/cm^3^ supplied by PetkimPetrochemical Company (Izmir, Türkiye). Natural huntite hydromagnesite mineral was obtained from the Tirtar district of Isparta, Türkiye. Stearic acid (C_17_H_35_COOH) was used as a surface modifier in coating the mineral powders. To achieve a synergistic effect, calcite and clay minerals were supplied by Anadolu Mikronize company in the Niğde region in Türkiye. For the preparation of the compounds, the ratios of mineral powders and polymer matrix were first determined. According to these ratios, different amounts of huntite hydromagnesite, clay, and calcite mixtures were prepared. The mixtures were mixed in a high-speed mixer at 2000 rpm for 10 min. The twin-screw extruder was fed from the extruder hopper at 20 rpm, and the main screw speed of the extruder was set to 190 rpm. The zone temperatures of the extruder were set to 180 °C, and the pressure was set to 10 bar. After the extrusion process, the composite pellets were dried in an oven, and the remaining moisture was removed. Following this process, PP composite pellets were melted in a high-speed laboratory thermokinetic machine at 2000 rpm for 25 s while stirring. The molten polymer exiting the machine was placed in the mold cavity between Teflon sheets, which were used to prevent direct contact of the PP composites with the hot press metal sheets during heating and pressing. Then, the sheets were first hot-pressed at 220 °C for 30 s using a Hydraulic Press Machine (Labtech Engineering, Thailand), followed by cold-pressing for 3 min. The formulation table in Table 1 shows the coding and ratios of the produced polypropylene composite samples. In this table and in the following, the following designations of components are used: PP—polypropylene, H—huntite/hydromagnesite, CA—calcite, CL—clay.

Our previous studies showed that a good degree of flame retardancy was achieved by using huntite and hydromagnesite at around 50–60% [19,20]. Therefore, as shown in the table, samples with huntite and hydromagnesite levels of 40% and 70% were used as the basis for the sample formulation. The mineral additive ratio in the mixture was kept constant, while efforts were made to reduce the levels of huntite and hydromagnesite within the minerals. This was achieved by using substitute inorganic minerals.

Morphological images of composite samples prepared with huntite, hydromagnesite, calcite, and clay mineral powders were obtained at different magnifications using a Carl Zeiss Sigma 300 VP scanning electron microscope (Carl Zeiss Microscopy GmbH, Jena, Germany)). The functional groups and bonds of the composites were determined using Fourier Transform Infrared Spectroscopy (FTIR) with a Bruker Alpha II spectrometer (Bruker Optik GmbH, Ettlingen, Germany). The analysis was performed in the wavelength range of 4000–500 cm^−1^ with a spectral resolution of 4 cm^−1^. The mechanical behavior of the composite samples was determined using a tensile testing machine (MTS Criterion) according to the ISO 527-2 standard [26]. Tensile strength was determined by testing with a 5 kN load cell and a test speed of 50 mm/min. To shed light on its flame-retardant properties, the thermal degradation behavior of the composites was evaluated using a TA Instruments TGA-SDT Q600 (TA Instruments, New Castle, DE, USA). The analysis was performed under a nitrogen atmosphere by heating the samples from room temperature to 800 °C at a heating rate of 10 °C/min. The flame-retardancy behavior of the composites was determined using both the UL-94 vertical burn test and the LOI test. Samples measuring 100 mm × 10 mm × 3 mm were used for the LOI test. UL-94 tests were performed by exposing samples measuring 125 mm × 13 mm × 3 mm to an open flame, in accordance with the standards specified in ASTM D3801 [27]. The time required for the flames to extinguish spontaneously was recorded. The LOI test was performed by measuring the minimum oxygen concentration required for sustained combustion. Mechanical and flame-retardancy tests were performed on at least n = 5 samples for each formulation. Three samples were tested for each additive and ratio.

## 3. Results and Discussion

**Figure 1:**
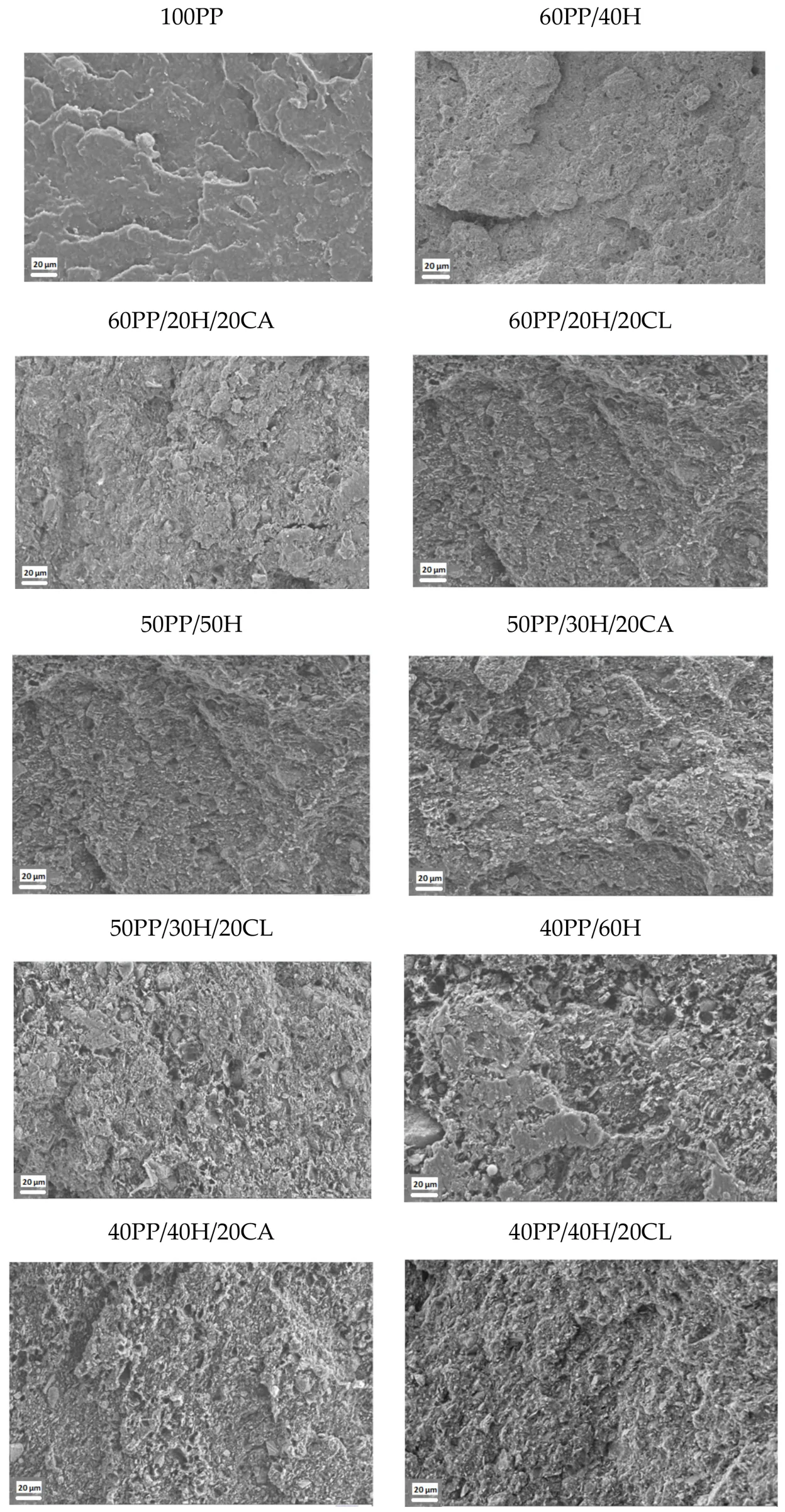
SEM analysis results of PP-, H-, CA-, and CL-filled PP composites.

Figure 1 shows SEM images of pure PP and mineral-reinforced composites. While the pure PP sample exhibited a smooth, continuous, and homogeneous surface morphology, the addition of mineral reinforcement significantly increased surface roughness. In composites containing H, it was determined that the mineral particles were generally homogeneously distributed within the polymer matrix, but the surface became more heterogeneous with increasing filler content. In particular, in hybrid composites where H was used together with calcite in the 60PP and 50PP series, the mineral phase was more evenly distributed within the matrix, and significant agglomeration formation was limited. In composites containing clay, however, the surface morphology exhibited a more irregular and finely rough structure. No significant macrovoids or serious porosity resulting from production were observed in any of the composites examined, indicating that the extrusion and injection processes ensured the successful distribution of mineral fillers within the matrix. The 30PP series, which failed to meet standard test conditions and showed premature fracture during the tensile test, was not included in the SEM analysis.

SEM images morphologically support the results obtained from mechanical tests. The increase in surface roughness and the widespread distribution of the mineral phase within the matrix with the addition of mineral admixture are consistent with the increase in elastic modulus observed in the composites. The literature also reports that in H-based polymer composites, the elastic modulus increases as a result of rigid mineral particles restricting the movement of polymer chains, and this effect is supported by homogeneous filler distribution [19,20,28]. Conversely, many studies have reported that high mineral content makes the surface morphology more heterogeneous and reduces the continuity of the polymer phase, contributing to a decrease in tensile strength and elongation at break [28,29,30]. In the present study, the observation of a more homogeneous filler distribution, especially in hybrid composites containing calcite, is consistent with the higher elastic modulus and tensile strength values obtained in the same formulations. This suggests that calcite can not only act as an auxiliary mineral but also contribute positively to mechanical performance by improving the distribution of H particles within the matrix. However, it is not possible to make definitive judgments about the strength or adhesion quality of the filler-matrix interface bond based solely on surface SEM images. Therefore, the current assessments are based on a combined interpretation of the observed morphological features and the mechanical results.

**Figure 2:**
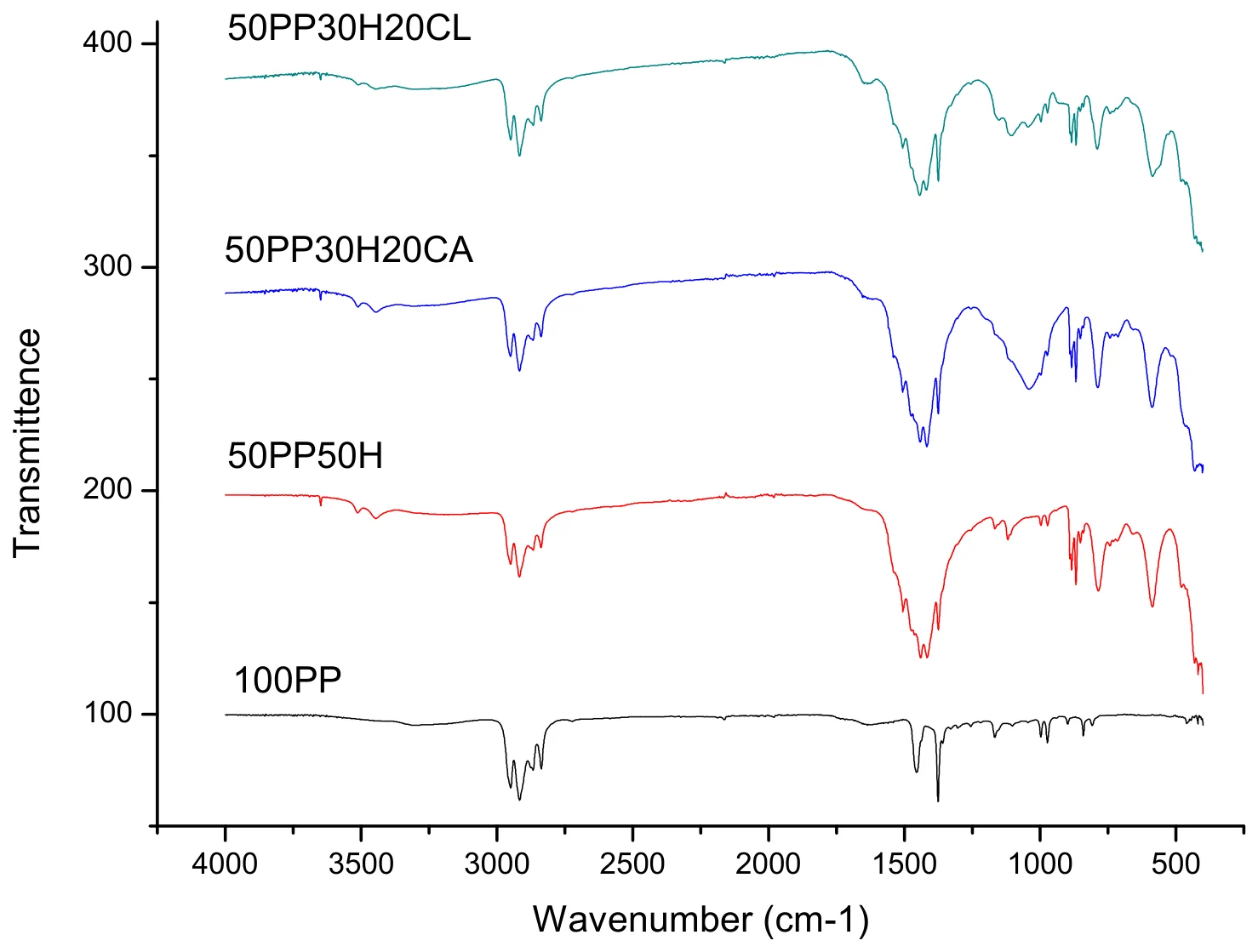
FTIR analysis of composites.

When the FTIR spectra were examined (Figure 2), it was determined that the bands observed in the regions of approximately 2950, 2915–2920 and 2835–2865 cm^−1^ in all samples correspond to the C–H stretching vibrations of the CH_3_ and CH_2_ groups in the PP chain, and the bands of approximately 1455 and 1375 cm^−1^ correspond to the CH_2_/CH_3_ deformation vibrations, respectively. In addition, absorptions in the regions of approximately 1165, 997–998, 972, and 840–842 cm^−1^, which are characteristic bands of PP, were clearly observed in the 100PP sample. In the 50PP/50H sample, while these PP bands are preserved, the widening OH band around 3400–3500 cm^−1^ and the prominence of bands around 1360 cm^−1^ indicate the contribution of hydroxyl and carbonate groups in the huntit/hydromagnesite structure. In the 50PP/30H/20CA sample, in addition to the PP and huntit/hydromagnesite bands, bands that become prominent in the regions of approximately 1410–1420, 875, and 710–713 cm^−1^ are associated with the carbonate vibrations of CaCO_3_ and are consistent with the characteristic FTIR regions of calcite. In the 50PP/30H/20CL sample, the strong band around 1000–1030 cm^−1^ and the prominence of bands between 900 and 920 cm^−1^ and 700–800 cm^−1^ indicate the contribution of Si–O and Al–OH vibrations in the clay structure. In general, it was observed that the addition of mineral additives did not eliminate the bands belonging to the basic functional groups of PP but rather added bands specific to the mineral phases to the spectrum, and the spectral structure changed significantly, especially in the 1000–1500 cm^−1^ range [31,32].

Based on the FTIR results, no clear evidence of the formation of new chemical bonds was observed, and the system is essentially a physical composite. The spectra show the retention of the characteristic vibrations of PP, along with the addition of the characteristic inorganic vibrations of hydromagnesite, calcite, and clay [19]. Stanienda [31] reported characteristic bands of PP around 2950, 2917, 2838, 1456, and 1376 cm^−1^ and identified bands of 1168, 997, and 841 cm^−1^ as characteristic vibrations of isotactic PP; the current 100PP spectrum is consistent with these findings. For huntit/hydromagnesite, the literature reports a broad OH band around 3450 cm^−1^, a water deformation band around 1620 cm^−1,^ and a carbonate band around 1350–1360 cm^−1^ as characteristic. Therefore, the changes observed in 50PP/50H and other H-containing composites support the structural contribution of huntit/hydromagnesite. García-Tojal et al. [32] and other mineralogical studies reported that calcite could be identified by bands of approximately 1415–1425, 875, and 712 cm^−1^; thus, the prominence of these bands in the 50PP/30H/20CA spectrum indicates that the addition of CA was successfully detected. In contrast, the emergence of Si–O stretching around 1000–1030 cm^−1^ and Al–OH vibrations around 910 cm^−1^ in the clay-containing sample is consistent with the values given in the literature for clay minerals. In conclusion, the FTIR spectra show that the additions of H, CA, and CL are incorporated into the spectral characteristics of the composites without altering the basic chemical structure of PP and that the four compositions can be distinguished from each other thanks to the unique vibrational bands of each mineral.

Table 2 presents the tensile test, elastic modulus, and elongation at break results of PP-based composites with different formulations. The elastic modulus of the reference sample, pure PP, was determined to be 1106 MPa, the maximum tensile stress 32.7 MPa, and the elongation at break 6.1%.

**Figure 3:**
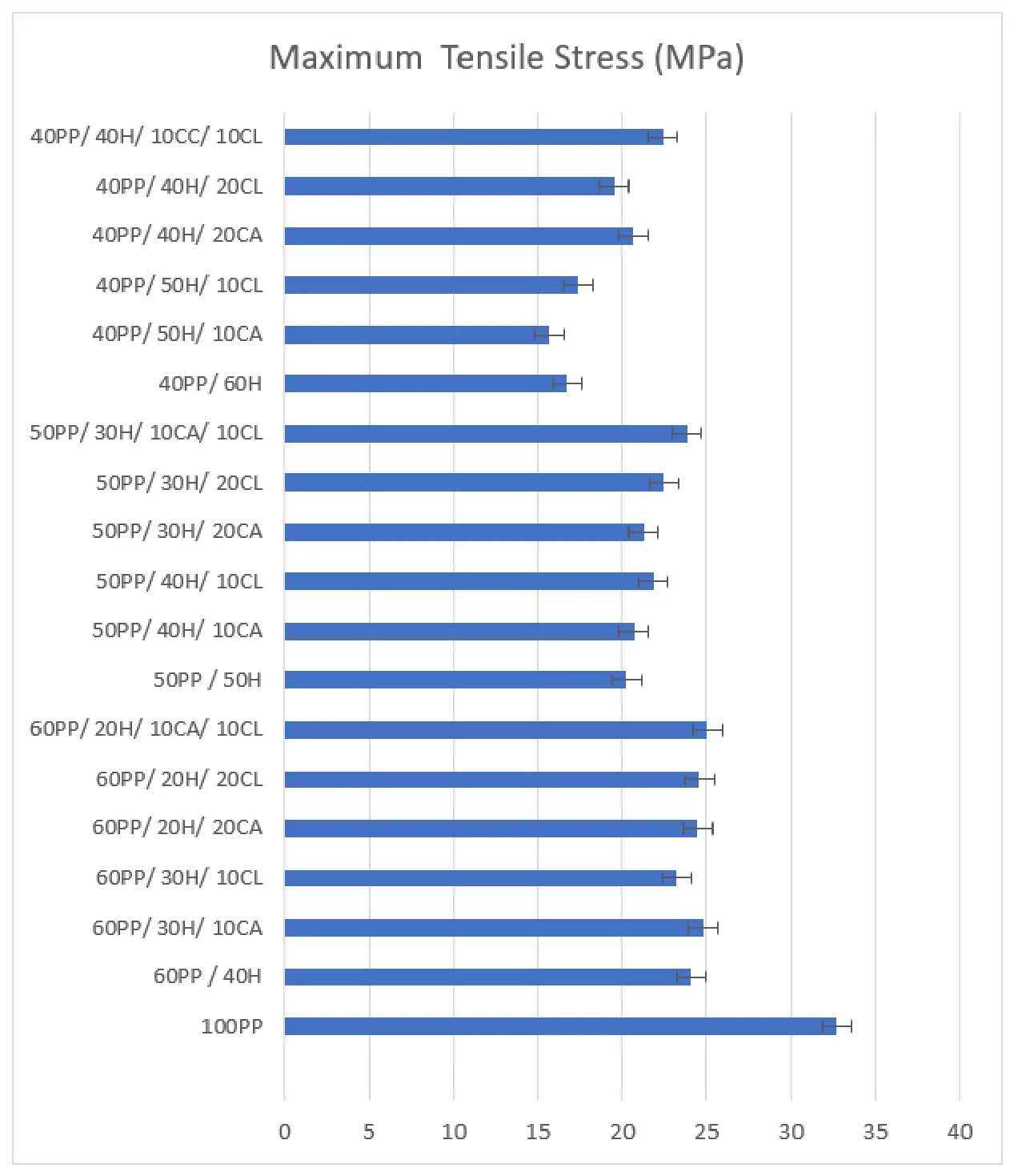
Tensile strength values of the composite samples.

For comparison purposes, tensile strength values are also discussed in Figure 3. Accordingly, the maximum tensile stress values in the composite samples are lower than those of the reference sample, and the measurable values ranged from 15.66 to 25.03 MPa. In the 60% PP series, the maximum tensile stress was observed in the range of 23.21–25.03 MPa. The highest value was obtained in the hybrid composite containing 20% H, 10% calcite, and 10% clay (25.03 MPa), while the lowest value was measured in the composite containing 30% H and 10% clay (23.21 MPa). In the 50% PP series, the maximum tensile stress ranged from 20.25 to 23.85 MPa. In this series, the highest tensile stress was obtained in the hybrid composite containing 30% H, 10% calcite, and 10% clay (23.85 MPa). The lowest value was determined in the composite containing 50% H (20.25 MPa). In the 40% PP series, maximum tensile stress values were measured in the range of 15.66–22.41 MPa. The highest value was obtained in the hybrid composite containing 40% H, 10% calcite, and 10% clay (22.41 MPa), while the lowest value was determined in the composite containing 50% H and 10% calcite (15.66 MPa). Premature fracture occurred in all composite samples of the 30PP series during tensile testing; therefore, maximum tensile stress values could not be determined. As observed in our earlier study, a significant decline in both tensile strength and elongation at break occurs when the polymer matrix content is reduced to 30%. The similar outcome encountered in the present study is consistent with these previous findings. One potential reason for this is the excessive agglomeration resulting from a high filler content, which in turn restricts chain mobility. Furthermore, a severe disruption in the continuity of the matrix serves as another significant factor contributing to this phenomenon.

**Figure 4:**
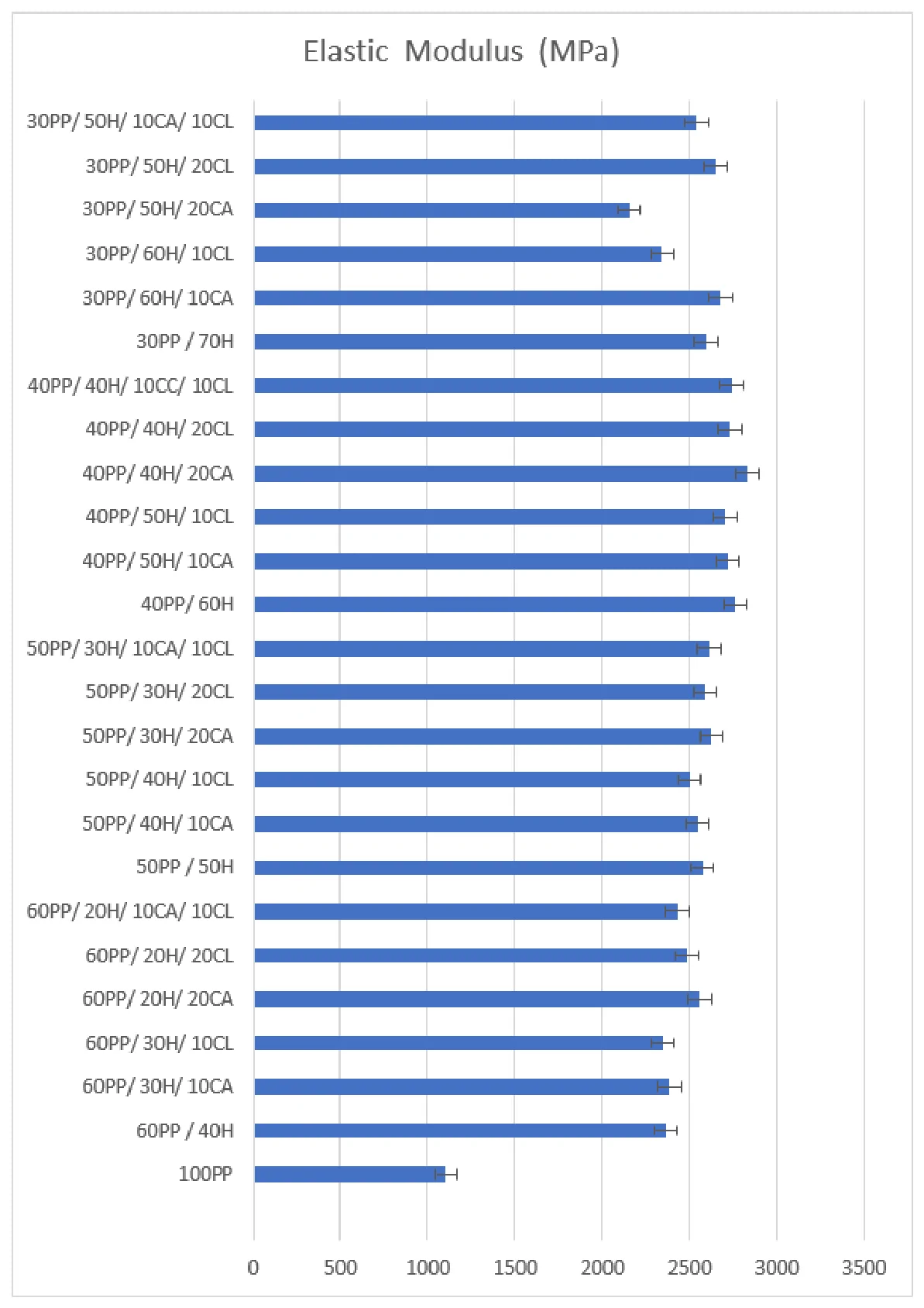
Elastic Modulus values of the composite samples.

Figure 4 compares the elastic modulus values of the samples. Accordingly, the elastic modulus values in all composite samples increased significantly compared to pure PP. In the series containing 60% PP, the elastic modulus values ranged from 2347 to 2561 MPa. In this series, the lowest elastic modulus value was obtained in the composite containing 30% H and 10% clay (2347 MPa), while the highest value was determined in the sample containing 20% H and 20% calcite (2561 MPa). In the hybrid composite containing 20% H, 10% calcite, and 10% clay, the elastic modulus was measured as 2431 MPa. In composites containing 50% PP, the elastic modulus values ranged from 2503 to 2629 MPa.

In this series, the highest elastic modulus value was obtained in the sample containing 30% H and 20% calcite (2629 MPa), while the lowest value was determined in the composite containing 30% H and 10% clay (2503 MPa). The hybrid composite containing 30% H, 10% calcite, and 10% clay exhibited a performance close to the highest values in this series, with an elastic modulus value of 2617 MPa. In the 40% PP series, elastic modulus values ranged from 2706 to 2836 MPa. The highest elastic modulus was obtained in the composite containing 40% H and 20% calcite (2836 MPa), while the lowest value was measured in the sample containing 50% H and 10% clay (2706 MPa). The elastic modulus of the hybrid composite containing 40% H, 10% calcite, and 10% clay was determined to be 2742 MPa. In the 30% PP series, elastic modulus values ranged from 2156 to 2679 MPa. In this series, the highest elastic modulus was obtained in the composite containing 60% H and 10% calcite (2679 MPa), while the lowest value was determined in the sample containing 50% H and 20% calcite (2156 MPa).

**Figure 5:**
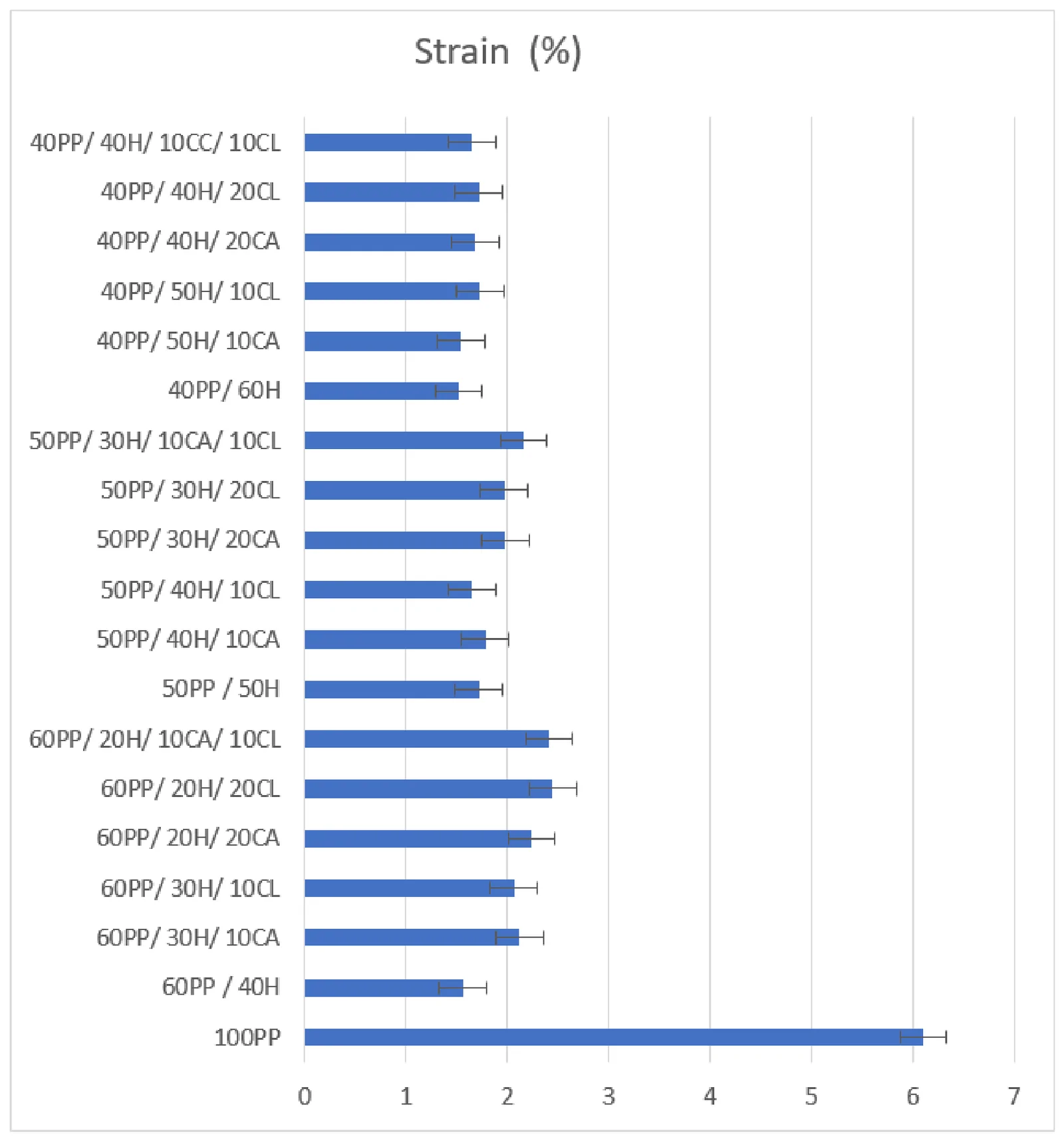
Elongation at break values of the composite samples.

The elongation at break values of the samples are discussed in Figure 5 for comparison purposes. As can be seen from the figure, the elongation at break values in the composite samples decreased significantly compared to the reference sample, and the measurable values ranged from 1.52% to 2.45%. In the 60% PP series, the elongation at break ranged from 1.56% to 2.45%. The highest value was obtained in the composite containing 20% H and 20% clay (2.45%), while the lowest value was determined in the composite containing 40% H (1.56%).

In the 50% PP series, the elongation at break values ranged from 1.65% to 2.16%. The highest elongation at break was measured in the hybrid composite containing 30% H, 10% calcite, and 10% clay (2.16%), while the lowest value was obtained in the composite containing 30% H and 10% clay (1.65%). In the 40% PP series, the elongation at break was determined to be in the range of 1.52–1.73%. The highest value was obtained in the composite containing 50% H and 10% clay (1.73%), while the lowest value was measured in the composite containing 60% H (1.52%). Since premature fracture occurred in all composite samples in the 30PP series during the tensile test, elongation at break values could not be determined in these samples.

When mechanical behavior is considered in general, the elastic modulus values of all mineral-reinforced composites increased compared to pure PP, while the maximum tensile stress and elongation at break decreased; in general, decreasing the PP ratio and increasing the total mineral filler content increased the rigidity of the composites while decreasing their ductility and tensile strength. The increase in elastic modulus can be explained by H and other mineral fillers limiting polymer chain movement and is consistent with previous studies [19,29,30]. In contrast, the decrease in tensile stress and elongation at break can be attributed to mineral fillers limiting the deformation capacity of the polymer matrix. When comparing composites with the same PP ratio, the fact that systems where H is used in hybrid form with calcite and clay exhibit higher elastic modulus, tensile strength, and elongation at break in most cases compared to composites containing only H supports the synergistic mechanical improvement reported by Atay et al. [22], which is important in hybrid mineral systems [33,34].

However, the 30PP series containing 70% total filler stood out significantly from other composites. While the expected increase in elastic modulus could not be achieved in this series, the maximum tensile stress and elongation at fracture could not be reliably determined due to the premature fracture of all specimens during the tensile test. Considering that Atay et al. [22] and Yücetürk et al. [28] successfully performed mechanical measurements up to 50–60% filler ratios, this behavior observed at the 70% filler level in the present study shows that at very high filler loadings, mechanical properties are strongly dependent not only on the total filler amount but also on microstructural factors such as particle distribution, filler-matrix interaction, and matrix continuity [35,36].

**Figure 6:**
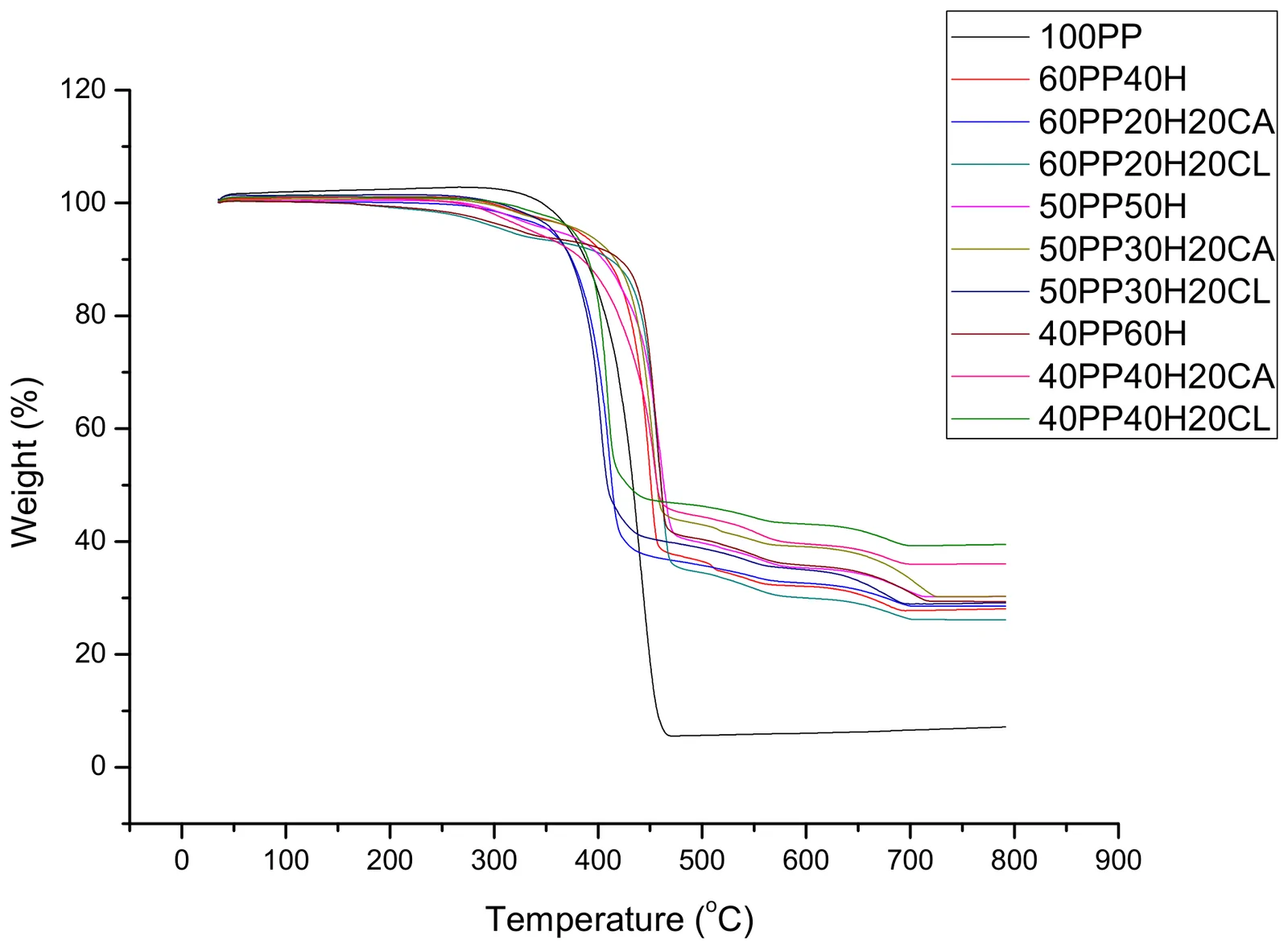
TGA curves of composite samples.

The thermal degradation behavior of the composites is illustrated in Figure 6. Before examining this, it is appropriate to evaluate the thermal behavior of the additives. Accordingly, the degradation of hydromagnesite occurs approximately between 220 °C and 550 °C. In the 200–250 °C range, crystalline water is released, while water from hydroxide groups is liberated between 380 °C and 450 °C. Around 510–550 °C, carbon dioxide (CO_2_) is released, leaving behind a solid residue of magnesium oxide (MgO). Huntite decomposes gradually, with significant activity in the 450–800 °C and 600–630 °C ranges. The carbonate ions bonded to magnesium atoms decompose, releasing CO_2_ gas and leaving behind calcium-based components and magnesium oxide. The final solid residue consists of magnesium oxide and calcium oxide (CaO). The decomposition of calcite begins in the range of approximately 600–700 °C and is rapidly completed above 850 °C, resulting in the release of CO_2_ and a solid residue of calcium oxide. Regarding the thermal degradation of clay, the removal of physical water is observed around 100–200 °C. In this initial stage, free water (adhesion water) present within the pores of the clay, as well as interlamellar water (water held between the clay layers), evaporates and is removed. Between 400 and 700 °C, the hydroxyl (-OH) groups present within the crystal lattice structure combine and depart from the structure as water vapor. Al-Si-O compounds are formed, and at higher temperatures, new phases emerge.

In this context, it is observed that the properties of the additives are reflected in the thermal behavior of the composites (Figure 6). The TGA plot reveals that the primary mass loss for all samples occurs within the range of approximately 350–470 °C. In the pure 100PP sample, degradation started at approximately 350 °C, the majority of the mass loss occurred in the range of 400–450 °C, and only 5–7% of the sample mass remained after approximately 450 °C. In mineral-reinforced composites, the degradation zone generally occurred in the range of 360–470 °C, and the amount of residue increased significantly at 800 °C. The residue amounts at 800 °C were determined to be approximately 36%, 34%, and 37% in the 60PP/40H, 50PP/50H, and 40PP/60H samples, respectively, while these values were approximately 31%, 30%, and 32% in the 60PP/20H/20CA, 50PP/30H/20CA, and 40PP/40H/20CA samples. In the clay-containing 60PP/20H/20CL, 50PP/30H/20CL, and 40PP/40H/20CL samples, the residue amounts at 800 °C were approximately 29%, 28%, and 30%. In general, reducing the PP content from 60% to 40% and increasing the mineral additive ratio significantly increased the amount of residue remaining at high temperatures compared to pure PP. Furthermore, the observation of a more gradual mass change between 450 and 700 °C after degradation in composites indicates that thermal transformations of mineral components at high temperatures contribute to the TGA behavior.

The results obtained are generally consistent with the TGA behavior reported in the literature for PP/mineral filler composites. Mapossa et al. [37] reported that in PP/CaCO_3_-based composites, pure PP degrades in a single step between approximately 340–480 °C, while in CaCO_3_-containing systems, additional degradation zones and significant residue formation occur at high temperatures; this is similar to the continued mass change and high residue amount in the composites after 450 °C in the present study. In the study conducted by Risite et al. [38] on PP/clay composites, it was stated that the addition of clay increased the degradation onset temperature and that this was related to the formation of a protective barrier that limited the diffusion of volatile products formed during polymer degradation. Similarly, Bogoeva-Gaceva et al. [39] showed that the addition of mineral fillers such as clay and talc to PP can increase the degradation temperature. Corcione et al. [40] reported that the amount of CaCO_3_ altered the thermal decomposition behavior of PP/LDPE-based systems and that the thermal decomposition kinetics changed with increasing filler ratio. Regarding huntit/hydromagnesite, a study on a huntit/hydromagnesite/PP system containing P_3_O_10_^5−^ reported that the addition of huntit/hydromagnesite increased the amount of residue and supported the formation of a more compact protective residue layer. Therefore, the high-temperature residues reaching 28–37% in the mineral-added samples in the present study, compared to the approximately 5–7% residue in pure PP, can be explained by the inorganic structures of H, CA, and CL and the mineral phases remaining at high temperatures. However, the increase in residue amount does not directly mean that the decomposition temperature increases proportionally; the amount of mineral additive, the thermal decomposition of the mineral itself, and the PP–filler interaction should be evaluated together.

The flame-retardancy results of the composite samples are given in Table 3. In the pure PP (100PP) sample, the cotton indicator ignited, no classification was obtained from the UL-94 test (NR), and the LOI value was determined to be 17.6%. It was determined that the cotton indicator did not ignite in all composites containing mineral additives.

In all samples in the 60PP series, the first burn time (t1) exceeded 30 s, and no sample received a UL-94 classification. In the 50PP series, only the 50PP/50H, 50PP/40H/10CA, and 50PP/30H/10CA/10CL samples met the UL-94 V1 classification, while the other samples were evaluated as NR. In this series, only the 50PP/50H sample had a determined LOI value, which was measured at 27%. In the 40PP series, samples 40PP/60H, 40PP/50H/10CA, 40PP/40H/20CL, and 40PP/40H/10CA/10CL met the UL-94 V0 rating, with the sample 40PP/50H/10CL being classified as NR. The shortest first burn time in this series was measured at 1 s in sample 40PP/60H. LOI values ranged from 31.2% to 37.4%, with the highest value of 37.4% obtained in sample 40PP/40H/10CA/10CL. All composites in the 30PP series met the UL-94 V0 rating. In this series, the first burn time ranged from 1 to 3 s, and the second burn time ranged from 3 to 6 s. LOI values were determined to be between 37.9% and 42.6%, with the highest LOI value of 42.6% obtained in the 30PP/60H/10CL sample. This was followed by 30PP/70H with 41.7%, 30PP/50H/10CA/10CL with 41.4%, 30PP/60H with 41.2%, 30PP/50H/20CA with 39.5%, and 30PP/50H/20CL with 37.9%, respectively.

In general, it was observed that flame-retardancy performance improved with decreasing PP content and increasing total mineral filler content. The UL-94 classification was determined as NR for the 60PP series, partially V1 for the 50PP series, largely V0 for the 40PP series, and V0 for all samples in the 30PP series. In parallel, LOI values also increased from 17.6% in pure PP to over 40% in composites containing 70% total mineral filler. A significant improvement in the flame-retardancy performance of composites was observed with decreasing PP content and increasing total mineral filler content. While no samples in the 60PP series received UL-94 classification, only some hybrid composites in the 50PP series reached class V1, and V0 classification was obtained in the 40PP and 30PP series, revealing the decisive effect of total filler content on flame retardancy. Similarly, the increase in LOI values from 17.6% in pure PP. Similarly, the increase in LOI values from 17.6% in pure PP to 42.6% in composites containing 70% total filler also supports this trend. These results are consistent with the reports by Atay and Çelik (2010, 2013) that flame retardancy improves with increasing H content [19,20]. Similarly, Zhang (2003), Fernandez (2009), and Hollingbery (2012) reported that mineral-based flame retardants improve UL-94 performance and oxygen index at high filler ratios [33,35,41]. The fact that the cotton indicator did not ignite in all mineral-reinforced composites in our study is consistent with these studies. However, the inability of samples containing 40% filler in the 60PP series to meet the UL-94 classification shows that, as emphasized in the literature, sufficient flame-retardant effect is not achieved below a certain critical filler ratio in H-based systems. When hybrid systems with the same total filler ratio are compared, it is seen that the addition of calcite and clay maintains or, in some cases, improves the flame-retardant performance compared to reference composites containing H. In particular, the fact that the 40PP/50H/10CA, 40PP/40H/20CA and 40PP/40H/10CA/10CL samples in the 40PP series achieved V0 classification and that the 40PP/40H/10CA/10CL composite showed the highest LOI value in the series with 37.4% reveals the positive effect of the hybrid mineral approach. Similarly, the fact that all composites in the 30PP series achieved V0 class and that the LOI values were in the range of 37.9–42.6% confirms the effectiveness of hybrid systems at high mineral loadings. While Yılmaz Atay et al. (2024) [22] reported that V0 classification could be obtained at lower H amounts in H/calcite/zeolite hybrid systems, Yücetürk et al. (2024) also showed that auxiliary minerals, by creating a synergistic effect with H, improve UL-94 and LOI performance [28]. Furthermore, Chen et al. (2013), Deng et al. (2014), and Fredi et al. (2019) reported that the combined use of different flame-retardant additives provides higher flame retardancy compared to mineral systems used alone [15,18,25]. Unlike the combinations of H and boric acid, zeolite, and IFR (intumescent flame retardant) commonly studied in the literature, the system used in the present study includes an H/calcite/clay hybrid structure. Therefore, the preservation of the V0 classification and the increase in LOI values above 40%, especially with the addition of clay, demonstrate that this hybrid mineral combination can be an effective alternative in PP composites.

As a limitation of this work, it can be noted that the studies were conducted for a limited set of ratios of mineral additives: calcite, huntite, hydromagnesite and clay. Only polypropylene was used as a base.

## 4. Perspectives on the Use of Artificial Intelligence for Composite Modelling

Although artificial intelligence algorithms and machine learning models were not used in the analytical process adopted in this study—where the behavior of the hybrid composites was assessed through direct laboratory measurements—computational tools represent a promising direction for materials engineering. The novelty of the presented H/CA/CL hybrid mineral system lies in achieving a UL-94 V0 rating and an LOI exceeding 40%, while the premature brittle fracture observed at 70 wt% total filler loading underscores a clear structural threshold. To address such experimental trade-offs without exhaustive destructive testing, predictive deep neural networks have been demonstrated to classify polymer fire behavior, forecasting LOI, UL-94 flammability ratings, and peak heat release rates [42]. Cloud-based analytical platforms, such as the module integrated into the MatVerse platform, further illustrate how chemical descriptors combined with synthetic data generators can accelerate the screening of low-flammability compositions prior to physical synthesis [43].

From a practical perspective, machine learning algorithms can specifically target the loss of polymer ductility associated with high mineral filler content by identifying optimal PP/H/CA/CL formulations that preserve structural integrity [28]. Because the selection and ratio of mineral phases fundamentally govern mechanical strength parameters [28,30], multi-input artificial neural networks (ANNs), Gaussian processes, and active learning loops utilizing multi-objective Bayesian optimization (MOBO) enable precise mapping of component mass fractions against Young’s modulus and flammability metrics [44,45]. Such algorithms establish exact filler loading thresholds, thereby preventing premature brittle failure without compromising tensile strength or flame retardancy. Furthermore, artificial neural networks trained on microscale combustion calorimetry and thermophysical data offer a robust paradigm for estimating heat release capacity, flammability properties, and elastic modulus in mineral-filled composites [46,47,48,49,50]. Integrating these computational approaches with empirical testing provides a consistent, evidence-based strategy to overcome high-filler brittleness while maintaining fire safety.

## 5. Conclusions

This study investigated the effects of hybrid mineral filler systems containing huntite/hydromagnesite, calcite, and clay on the mechanical and flame-retardant properties of polypropylene composites. The findings showed that increasing the total mineral filler ratio significantly improved the rigidity and flame-retardant performance of the composites, but led to a decrease in ductility and tensile strength at higher filler ratios. Specifically, the elastic modulus increased from 1106 MPa in neat PP to a peak value of 2836 MPa in the 40PP/40H/20CA composite due to restricted chain mobility, whereas maximum tensile stress dropped to a range of 15.66–25.03 MPa and strain at break decreased to 1.52–2.45%. The use of H in combination with calcite and clay contributed to better preservation of mechanical properties compared to systems containing only H, and supported improved UL-94 and LOI performance, especially under high mineral loadings.

SEM morphological observations confirmed that the analyzed calcite particles improved dispersion and reduced agglomeration, while the incorporation of clay into the ternary systems enabled improved performance compared with the corresponding binary PP/H formulations. FTIR spectra confirmed the presence of predominantly physical interactions. The TGA char residue at 800 °C reached 28–37%, compared with only 5–7% for neat PP. Highly filled formulations in the 40PP series achieved UL-94 V-0 ratings, with LOI values reaching 37.4% for 40PP/40H/10CA/10CL. In contrast, all formulations in the 30PP series attained UL-94 V-0 ratings, with exceptionally high LOI values of up to 41.4% for 30PP/50H/10CA/10CL and 42.6% for 30PP/60H/10CL. However, the 70 wt% total filler loading in the 30PP series represents a structural threshold, as premature brittle fracture at the grips prevented standard tensile testing. Overall, the H/CA/CL hybrid mineral system has demonstrated the potential to strike an appropriate balance between mechanical performance and fire safety while providing halogen-free flame retardancy in PP composites. Offering advantages over the binary combinations found in the existing literature, these ternary combination systems are considered a promising alternative for applications that require high fire resistance.

The achievement of a UL-94 V-0 flammability rating and high LOI values makes these halogen-free formulations suitable for a wide range of applications. Potential examples include electrical equipment housings, protective sheathing for power cables, and protective enclosures for electronic systems exposed to thermal loads, offering the potential to replace halogen-containing polymers with safer structural materials. Their implementation and application will contribute to both the greening of individual industries and the achievement of the Sustainable Development Goals in general.

Research focused on further reducing the content of huntite hydromagnesite through the addition of different agents into the composites is considered promising; this is expected to ensure a sufficient level of flame-retardant performance and reduce deterioration of mechanical properties. Specifically, upcoming investigations will focus on incorporating synergistic agents and surface modifiers to retain the UL-94 V0 classification and LOI values above 40% while resolving the premature brittle failure at extreme filler ratios.

## Figures and Tables

**Table 1:** Formulations of the composite samples.

No	Sample	PP (%)	H (%)	CA (%)	CL (%)
1	100PP	100	-	-	-
2	60PP/40H	60	40	-	-
3	60PP/30H/10CA	60	30	10	-
4	60PP/30H/10CL	60	30	-	10
5	60PP/20H/20CA	60	20	20	-
6	60PP/20H/20CL	60	20	-	20
7	60PP/20H/10CA/10CL	60	20	10	10
8	50PP/50H	50	50	-	-
9	50PP/40H/10CA	50	40	10	-
10	50PP/40H/10CL	50	40	-	10
11	50PP/30H/20CA	50	30	20	-
12	50PP/30H/20CL	50	30	-	20
13	50PP/30H/10CA/10CL	50	30	10	10
14	40PP/60H	40	60	-	-
15	40PP/50H/10CA	40	50	10	-
16	40PP/50H/10CL	40	50	-	10
17	40PP/40H/20CA	40	40	20	-
18	40PP/40H/20CL	40	40	-	20
19	40PP/40H/10CA/10CL	40	40	10	10
20	30PP/70H	30	70	-	-
21	30PP/60H/10CA	30	60	10	-
22	30PP/60H/10CL	30	60	-	10
23	30PP/50H/20CA	30	50	20	-
24	30PP/50H/20CL	30	50	-	20
25	30PP/50H/10CA/10CL	30	50	10	10

**Table 2:** Mechanical behaviours of the composite samples.

No	Sample	Elastic Modulus (MPa)	*Std Dev* *	Maximum Tensile Stress (MPa)	*Std Dev* *	Elongation at Break (%)	*Std Dev* *
1	100PP	1106	10.85	32.7	0.08	6.1	0.02
2	60PP/40H	2365	115.62	24.08	0.25	1.56	0.08
3	60PP/30H/10CA	2389	78.16	24.80	0.20	2.12	0.09
4	60PP/30H/10CL	2347	98.27	23.21	0.16	2.06	0.08
5	60PP/20H/20CA	2561	120.74	24.46	0.54	2.24	0.09
6	60PP/20H/20CL	2489	135.54	24.57	0.37	2.45	0.05
7	60PP/20H/10CA/10CL	2431	89.21	25.03	0.5	2.41	0.06
8	50PP/50H	2576	100.56	20.25	0.42	1.72	0.06
9	50PP/40H/10CA	2548	122.25	20.66	0.21	1.78	0.08
10	50PP/40H/10CL	2503	137.46	21.81	0.81	1.65	0.08
11	50PP/30H/20CA	2629	105.68	21.24	0.65	1.98	0.11
12	50PP/30H/20CL	2591	98.65	22.46	0.64	1.97	0.04
13	50PP/30H/10CA/10CL	2617	179.42	23.85	0.72	2.16	0.07
14	40PP/60H	2765	156.85	16.71	0.87	1.52	0.08
15	40PP/50H/10CA	2722	182.64	15.66	0.56	1.54	0.11
16	40PP/50H/10CL	2706	152.25	17.37	0.66	1.73	0.09
17	40PP/40H/20CA	2836	135.65	20.64	0.91	1.68	0.12
18	40PP/40H/20CL	2735	122.78	19.54	0.62	1.72	0.08
19	40PP/40H/10CA/10CL	2742	185.89	22.41	0.78	1.65	0.07
20	30PP/70H	2406	196.32	-		-	
21	30PP/60H/10CA	2679	205.78	-		-	
22	30PP/60H/10CL	2346	157.82	-		-	
23	30PP/50H/20CA	2156	211.69	-		-	
24	30PP/50H/20CL	2653	180.31	-		-	
25	30PP/50H/10CA/10CL	2542	232.41	-		-	

* *Std Dev*: standard deviation.

**Table 3:** Flame retardant behaviours of the composite samples.

No	Sample	1st Burning Time (t1) (s)	2nd Burning Time (t2) (s)	Cotton Indicator Ignited	UL-94 Rating *	LOI Value (%)
1	100PP	>30	-	Yes	NR	17.6
2	60PP/40H	>30		No	NR	-
3	60PP/30H/10CA	>30		No	NR	-
4	60PP/30H/10CL	>30		No	NR	-
5	60PP/20H/20CA	>30		No	NR	-
6	60PP/20H/20CL	>30		No	NR	-
7	60PP/20H/10CA/10CL	>30		No	NR	-
8	50PP/50H	4	15	No	V1	27
9	50PP/40H/10CA	7	19	No	V1	-
10	50PP/40H/10CL	>30	-	No	NR	-
11	50PP/30H/20CA	>30	-	No	NR	-
12	50PP/30H/20CL	>30	-	No	NR	-
13	50PP/30H/10CA/10CL	15	15	No	V1	-
14	40PP/60H	1	4	No	V0	36.3
15	40PP/50H/10CA	6	10	No	V0	32.9
16	40PP/50H/10CL	4	>30	No	NR	36.8
17	40PP/40H/20CA	2	>30	No	NR	31.2
18	40PP/40H/20CL	3	7	No	V0	33.6
19	40PP/40H/10CA/10CL	3	4	No	V0	37.4
20	30PP/70H	2	6	No	V0	41.7
21	30PP/60H/10CA	3	4	No	V0	41.2
22	30PP/60H/10CL	3	3	No	V0	42.6
23	30PP/50H/20CA	3	5	No	V0	39.5
24	30PP/50H/20CL	1	4	No	V0	37.9
25	30PP/50H/10CA/10CL	2	3	No	V0	41.4

* NR: not rated.

## Data Availability

The original contributions presented in this study are included in the article. Further inquiries can be directed to the corresponding authors.
